# Regeneration of full-thickness skin defects by differentiated adipose-derived stem cells into fibroblast-like cells by fibroblast-conditioned medium

**DOI:** 10.1186/s13287-017-0520-7

**Published:** 2017-04-20

**Authors:** Woojune Hur, Hoon Young Lee, Hye Sook Min, Maierdanjiang Wufuer, Chang-won Lee, Ji An Hur, Sang Hyon Kim, Byeung Kyu Kim, Tae Hyun Choi

**Affiliations:** 1 Department of Plastic and Reconstructive Surgery, Institute of Human–Environment Interface Biology, Seoul National University College of Medicine, Seoul, 110-799 Republic of Korea; 2 Biomedical Research Institute, Seoul National University Hospital, Seoul, 110-744 Republic of Korea; 3 Department of Preventive Medicine, Graduate School of Public Health, Seoul National University, Seoul, 152-742 Republic of Korea; 4 Department of Naval Architecture and Ocean Engineering, College of Engineering, Seoul National University 110-744, Seoul, Republic of Korea; 5 Department of Internal Medicine, School of Medicine, Yeungnam University, Daegu, 712-749 Republic of Korea; 6 Department of Internal Medicine, Keimyung University Dongsan Medical Center, Daegu, 700-712 Republic of Korea

**Keywords:** Human adipose-derived stem cell-derived conditioned medium, Type I collagen, Wound healing, Cell transplantation

## Abstract

**Background:**

Fibroblasts are ubiquitous cells in the human body and are absolutely necessary for wound healing such as for injured skin. This role of fibroblasts was the reason why we aimed to differentiate human adipose-derived stem cells (hADSCs) into fibroblasts and to test their wound healing potency. Recent reports on hADSC-derived conditioned medium have indicated stimulation of collagen synthesis as well as migration of dermal fibroblasts in wound sites with these cells. Similarly, human fibroblast-derived conditioned medium (F-CM) was reported to contain a variety of factors known to be important for growth of skin. However, it remains unknown whether and how F-CM can stimulate hADSCs to secrete type I collagen.

**Methods:**

In this study, we obtained F-CM from the culture of human skin fibroblast HS27 cells in DMEM media. For an in-vivo wound healing assay using cell transplantation, balb/c nude mice with full-thickness skin wound were used.

**Results:**

Our data showed that levels of type I pro-collagen secreted by hADSCs cultured in F-CM increased significantly compared with hADSCs kept in normal medium for 72 h. In addition, from a Sircol collagen assay, the amount of collagen in F-CM-treated hADSC conditioned media (72 h) was markedly higher than both the normal medium-treated hADSC conditioned media (72 h) and the F-CM (24 h). We aimed to confirm that hADSCs in F-CM would differentiate into fibroblast cells in order to stimulate wound healing in a skin defect model. To investigate whether F-CM induced hADSCs into fibroblast-like cells, we performed FACS analysis and verified that both F-CM-treated hADSCs and HS27 cells contained similar expression patterns for CD13, CD54, and CD105, whereas normal medium-treated hADSCs were significantly different. mRNA level  analysis for Nanog, Oct4A, and Sox2 as undifferentiation markers and vimentin, HSP47, and desmin as matured fibroblast markers supported the characterization that hADSCs in F-CM were highly differentiated into fibroblast-like cells. To discover the mechanism of type I pro-collagen expression in hADSCs in F-CM, we observed that phospho-smad 2/3 levels were increased in the TGF-β/Smad signaling pathway. For in-vivo analysis, we injected various cell types into balb/c nude mouse skin carrying a 10-mm punch wound, and observed a significantly positive wound healing effect in this full-thickness excision model with F-CM-treated hADSCs rather than with untreated hADSCs or the PBS injected group.

**Conclusions:**

We differentiated F-CM-treated hADSCs into fibroblast-like cells and demonstrated their efficiency in wound healing in a skin wound model.

## Background

Loss of the dermis in extensive full-thickness wounds such as burns, massive avulsion injuries, septic skin necrosis, or extended excision of scars, not completely solved by the application of split-thickness autografts, poses a serious problem [1, 2]. Conventional split-thickness skin grafts, often widely meshed and expanded, are utilized to close large wound deficits [3].

Recently, it has been recognized that fibroblasts can play an important role in skin regeneration and augmenting healing of wounds through their implantation [4]. However, for autogenic cells, being present in insufficient numbers limits their usage. As an alternative, allogeneic cells pose ethical and safety issues when considered for skin wound repair [5]. After full-thickness dermal injuries it is important to have an effective dermal replacement, because dermal tissue does not regenerate into normal dermis in vivo [6]. For these reasons, although still in the research stage, cells offer a more favorable choice for skin regeneration or wound healing than transplantation of dermal substitutes [4, 7], and stem cell therapy is clinically being investigated as a safe and effective method for repair of several types of tissue damage [8–10].

Wound healing is a well-orchestrated process that can be divided into three overlapping phases, beginning with the inflammatory phase, followed by the proliferative phase, and concluding with the remodeling phase [11]. Several cell types of mesenchymal origin have been implicated in these processes. These include fibroblasts, fibrocytes, and myofibroblasts that play critical roles in both early and late phases, where they contribute to the wound contraction, collagen deposition, and finally fibrosis [12, 13]. Collagens are extracellular matrix (ECM) proteins that are found in nearly all eukaryotic organisms except for plants and protozoa [14]. There are approximately 27 different types of collagens that have been identified, with type I collagen being the most prevalent; type I collagen is found in vertebrate connective tissues such as tendons, ligaments, bone, skin, and the cornea of the eyes [15].

Recently, articles concerning human adipose-derived stem cell-derived conditioned medium (hADSC-CM) have reported on its ability to stimulate collagen synthesis as well as migration of dermal fibroblasts. hADSCs are known to promote wound healing mainly through a paracrine mechanism, and it is plausible that ADSCs may exert their effect by secreting cytokines and growth factors that act on neighboring cells to repair the damaged tissues [16, 17]; thus the enriched media from these cells may be a good source of tissue repair factors. As such, hADSC-CM stimulated both collagen synthesis and migration of dermal fibroblasts, which improved the appearance of wrinkles and accelerated wound healing in animal models [18–20].

Similarly, human fibroblast (HS27)-derived conditioned medium (F-CM) was reported to contain a variety of factors known to be important in the growth of skin [21–23], and from research on hADSCs it is still not clear how F-CM stimulates hADSCs to secrete type I collagen. Our study goals were to investigate whether F-CM-treated hADSCs could be efficient in wound healing in an in-vitro model, and whether regeneration of full-thickness skin defects could be achieved by having the hADSCs transdifferentiate into fibroblast-like cells by F-CM in vivo.

## Methods

### hADSC isolation and culture

Discarded tissue from the abdomen was collected as skin and fat samples from Seoul National University Hospital (SNUH) with the written consent from the patients prior to surgery. The study was approved by the SNUH Ethics Committee (approval IRB No. 1108-098-374).

Human subcutaneous adipose tissue samples were separated from blood vessels, hair, and excess fat, and cut into small pieces prior to digestion with collagenase type I (Sigma-Aldrich, St. Louis, MO, USA) under gentle agitation for 1 h at 37 °C. The samples were then filtered with 70-μm mesh filters, and mixed with low-glucose DMEM supplemented with 10% fetal bovine serum (FBS; Gibco/Thermo Fisher, Waltham, MA, USA) and 1% penicillin/streptomycin solution (P/S; Gibco), and then centrifuged at 300 × *g* for 20 min. The ADSC fraction was washed with Hank’s balanced salt solution (HBSS) and centrifuged at 300 × *g* for 10 min; the supernatant was discarded. The cell pellet was resuspended in DMEM supplemented with 10% FBS and cultured in a humidified 5% CO_2_, 37 °C incubator. The culture medium was changed every 2 days.

### Preparation of F-CM

To obtain F-CM, human skin fibroblast HS27 cells (CRL-1634, 5 × 10^5^ cells; ATCC, Manassas, VA, USA) were cultured in high-glucose DMEM (Invitrogen-Gibco/Thermo Fisher, Waltham, MA, USA) supplemented with 10% FBS and 1% P/S. After reaching 80% confluency, the normal grown medium was discarded and the cells were washed twice with phosphate-buffered saline (PBS; 3 M, USA). Serum-free high-glucose DMEM supplemented with 1% P/S was added to HS27 cells and the cells were continued for culture at 37 °C and in a humidified atmosphere containing 5% CO_2_. After incubation for 2 days, the culture medium was collected and centrifuged at 300 × *g* for 5 min, and then filtered through a 0.2-μm syringe filter (Millipore, Billerica, MA, USA) for later use. The cell experiments were carried out with early passage (passage 1–5) cells.

### Western blot analysis

To observe protein level changes in hADSCs, HS27 cells, and F-CM-treated ADSCs (passages 2–5) after the differentiation treatment for 72 h, the cells were harvested in 200 μl of 1× RIPA buffer (40 mM Tris–HCl pH 7.4, 1% Triton X-100, 0.1% SDS, 0.15 M NaCl, 10% glycerol, 1 mM EDTA, 50 mM NaF, 20 mg/ml of 1 mM PMSF, 1 mM Na_3_VO_4_, 5 mM dithiothreitol, 1 μg/ml leupeptin, 1 μg/ml pepstatin, and 1 μg/ml aprotinin). In brief, the cell lysates were ultrasonicated in a sonicator bath and were centrifuged for 10 min at 10,000 × *g* at 4 °C. The protein concentrations were determined by BCA protein assay kit (Pierce, Rockford, IL, USA). Protein samples at 50 μg were separated by 8–12% SDS–polyacrylamide gel electrophoresis in each group and were transferred onto PVDF membranes. The membranes were then washed twice with Tris-buffered saline (pH 7.5, 10 mM Tris, 150 mM NaCl containing 0.1% Tween-20) (TBST) and blocked with 5% nonfat dried skim milk in TBST for 1 h at room temperature. They were then incubated with the primary antibodies (Table 1) overnight at 4 °C. The membranes were then washed with TBST and were continuously incubated with the appropriate horseradish peroxidase-conjugated secondary antibodies such as anti-mouse IgG or anti-rabbit IgG, and were developed in the ECL reagent (Santa Cruz, Dallas, TX, USA). The blots were reacted with chemiluminescence substrate (ECL; Millipore) and exposed to X-ray film. To calculate the western blot band, we used Image J (2.0; National Institute of Health, Bethesda, MD, USA) and GraphPad Prism (version 5.01; GraphPad, La Jolla, CA, USA).

**Table 1:** Antibody information for western blot analysis

Number	Antibody	Antibody size (kDa)	Dilution	Supplier
1	TGF-β	13	1:2000	Santa Cruz, USA
2	FGF2	19	1:4000	Cell Signaling Technology, USA
3	VEGF	21	1:1000	Santa Cruz, USA
4	Phospho-smad 2	60	1:500	Cell Signaling Technology, USA
5	Phospho-smad 3	52	1:500	Cell Signaling Technology, USA
6	Total-smad 2/3	60/52	1:1000	Cell Signaling Technology, USA
7	Smad 4	70	1:1000	Cell Signaling Technology, USA
8	Pro-collagen type I	170	1:2000	Santa Cruz, USA
9	B-actin	45	1:2000	Cell Signaling Technology, USA

### Sircol collagen assay

The manufacturer’s protocol was followed for the Sircol collagen assay (SCA) (Biocolor Life Science Assays, UK). In brief, 200 μl of conditioned medium was added to 1 ml of the colorimetric reagent and centrifuged for 30 min at 5000 × *g* for 20 min. The sample was released from the pellet with an alkali reagent (1.5 N NaOH) and was measured at 500 nm on a high-performance monochromator multimode microplate reader (BMG Labtech, Offenburg, Germany). Absolute values were obtained with a standard graph composed from collagen type I standard, which was supplied with the kit in the range of 5–100 μg/ml.

### FACS analysis

FACS analysis was performed for characterization of hADSCs, HS27 cells, and F-CM-treated ADSCs after differentiation for 72 h. The cells in each group were incubated with FITC-conjugated antibodies for CD14, CD19, CD34, CD45, and CD105 and PE-conjugated antibodies for CD13, CD54, and CD73 (all from BD Pharmingen/BD, Franklin Lakes, NJ, USA) for 30 min at room temperature. As control, the cells were stained with FITC-isotype control IgG and PE-isotype control IgG (BD Pharmingen). Cells were subsequently washed twice with FACS buffer and analyzed on a FACScan flow cytometer (Becton Dickson, Franklin Lakes, NJ, USA) using the CellQuest Pro software (Becton Dickson).

### RT-PCR analysis

To observe changes in the message levels of various differentiation markers after F-CM treatment, total RNA was isolated with the RNeasy mini kit (Qiagen, Germantown, MD, USA) according to the manufacturer’s instruction. Total RNA concentrations were determined by a UV–Vis spectrophotometer with absorbance at 260 nm (NanoDrop 2000; Thermo Scientific, Waltham, MA, USA). For RT-PCR, 1 μg of mRNA was reverse-transcribed with oligo(dT)18 using a thermal cycler (C-1000 Touch; Bio-Rad, Hercules, CA, USA). PCR was performed with a reaction mixture containing Taq DNA polymerase (Thermo Scientific) and the appropriate primers (Table 2) with a thermal cycler (C-1000 Touch; Bio-Rad). The amplification parameters were an initial 95 °C incubation for 5 min, followed by 40 amplification cycles of sequentially 95 °C for 30 sec, 53–58 °C for 30 sec, and 72 °C for 1 min. The reactions ended with 72 °C for 7 min, followed by 4 °C overnight.

**Table 2:** Primers for RT-PCR analysis

Number	Oligo name	Sequence (5′–3′)	Primer size
1	Nanog	Forward: CCT CCT CCC ATC CCT CAT	18
		Reverse: GGA TGG GCA TCA TGG AAA	18
2	Oct4	Forward:CCC TCC AGG TGG TGG	15
		Reverse:GGC CTT GGAAGC TTA	15
3	Sox2	Forward: CTC CGG GAC ATG ATC AGC	18
		Reverse: AGT GCT GGG ACA TGT GAA	18
4	Vimentin	Forward: AGG ATG TTG ACAATG CGT	18
		Reverse: ATC GAT TTG GAC ATG CTG	18
5	HSP47	Forward: AGC AGC AAG CAG CAC TAC	18
		Reverse: AAT TTC TCG TCC CAG TGT	18
6	Desmin	Forward: CAG ACC TAC TCT GCC CTC	18
		Reverse: GAT CAT CAC CGT CTT CTT	18
7	B-actin	Forward: TCC ACA TGC TTT ATT CCA	18
		Reverse: TGG CAC CCA GCA CAA TGA	18

### Immunohistochemistry

For staining collagen, tissue sections were cut and mounted on slides. Using the Discovery XT Automated Immunohistochemistry Stainer (Ventana Medical Systems Inc., Tucson, AZ, USA), the slides were incubated in DAB + H_2_O_2_ substrate for 8 min at 37 °C, followed by hematoxylin/Bluing Reagent counterstain at 37 °C. Reaction buffer (pH 7.6 Tris buffer) was also used for washing. The 12-μm thick sections were mounted on slides precoated with 10% poly-l-lysine (Sigma-Aldrich), and were blocked with goat serum for 1 h, followed by incubation with primary rabbit antibodies to anti-collagen type I (rabbit polyclonal, 1:40; Sigma-Aldrich) at 4 °C overnight. After washing with PBS, the sections were allowed to incubate with secondary antibodies conjugated with fluorochrome TRITC (goat anti-rabbit, 1:250; Sigma-Aldrich) at room temperature in the dark for 2 h. Following further PBS washes, the sections were mounted in fluorescent mounting medium and observed under a confocal microscope.

### Full-thickness skin wound model and cell transplantation

In the in-vivo wound healing assay using cell transplantation, balb/c nude mouse (male; 11 weeks old; weighing 20 ± 3 g) were fed and maintained under a 12-h light/dark cycle at 22–25 °C with 55–60% humidity. All of the conditions including diet, drinking, and defecation were normal. All experimental protocols were approved in strict accordance with the guidelines for the SNUH Institutional Animal Care and Use Committee (IACUC No. 13-0292-C0A3).

**Fig. 1:**
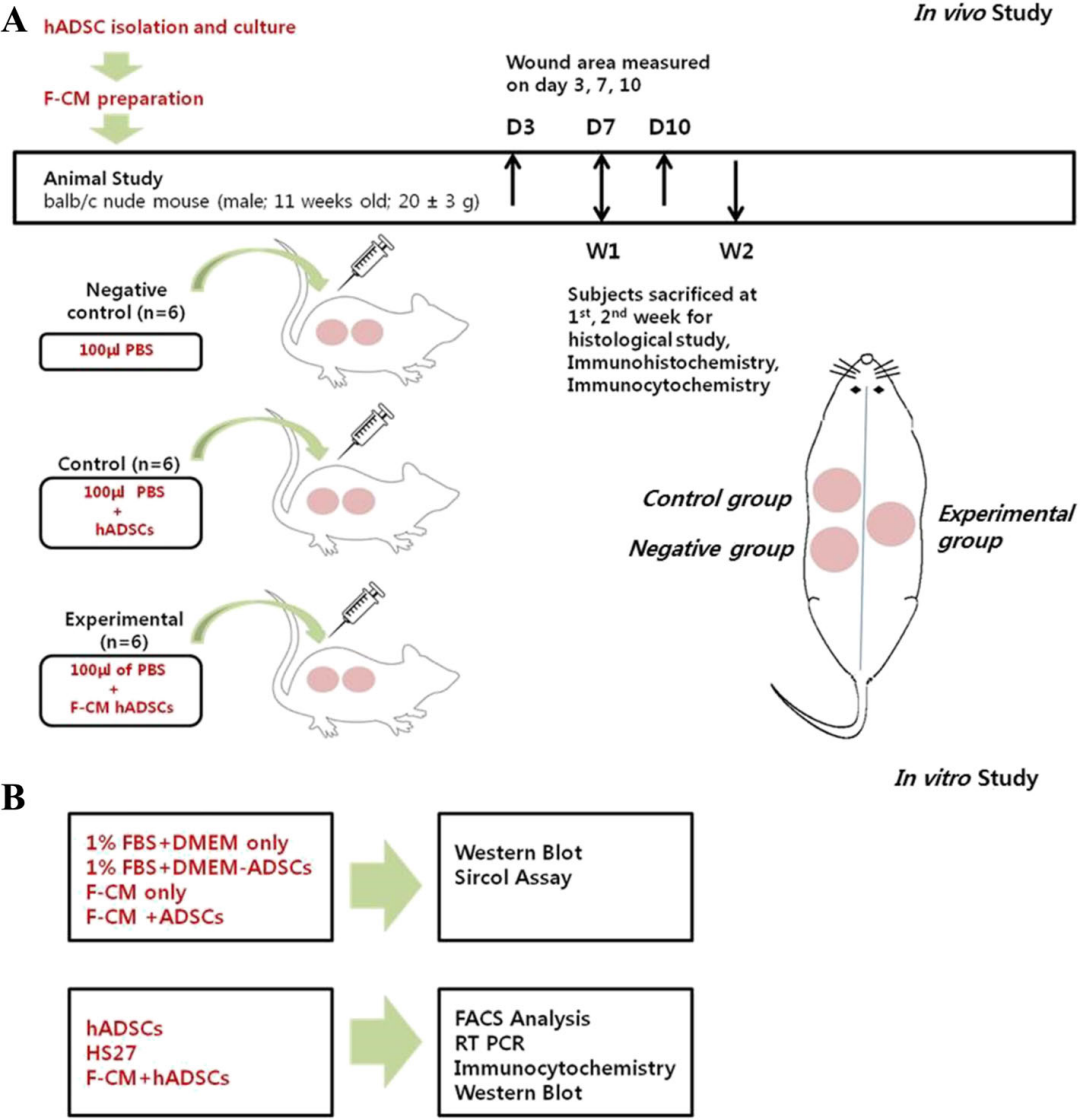
**a** In-vivo study design. **b** In-vitro study design. *F-CM* fibroblast-derived conditioned medium, *hADSC* human adipose-derived stem cell, *PBS* phosphate-buffered saline

For the full-thickness skin wound model, we anesthetized the mice with a subcutaneous injection of mixture of zoletil (0.1 ml/kg) and rompun (0.05 ml/kg). A full-thickness skin wound was produced on the dorsum by three 10-mm biopsy punches lateral to the midline of the back. After creation of the wounds, all mice were housed separately. In the negative control group (*n* = 6), 100 μl of PBS was injected intradermally around the wound base at six or seven injection sites (Fig. 1a, b). The control group (*n* = 6) received an intradermal injection with 100 μl of PBS containing 6 × 10^5^ hADSCs around the wound base at six or seven injection sites. The experimental group (*n* = 6) involved each mouse being intradermally injected with 100 μl of PBS containing 6 × 10^5^ F-CM-treated hADSCs around the wound base at six or seven injection sites.

### Wound healing analysis

The wounds for each individual mouse were photographed digitally, and the wound area was measured on days 3, 7, and 10 after cell transplantation. Time to wound closure was defined as the time at which the wound bed was completely reepithelialized and filled with new tissue. Wound area was measured by tracing the wound margin and calculated using an image analysis program (Image J; National Institute of Health). The investigators who measured the wound were blinded. The wound healing rate was calculated as follows [23]:

$$ \left(\mathrm{Area}\ \mathrm{of}\ \mathrm{original}\ \mathrm{wound}\ \hbox{--}\ \mathrm{area}\ \mathrm{of}\ \mathrm{remaining}\ \mathrm{wound}\right)\ /\ \mathrm{area}\ \mathrm{of}\ \mathrm{original}\ \mathrm{wound} \times 100. $$

### Histological study

To evaluate whether injected F-CM-treated cells enhanced wound healing in a full-thickness skin wound by repair involving a dense cell structure and secreted collagen, we sacrificed the animals 12 and 24 weeks after cell injection. The specimens were fixed in 10% buffered neutral formalin (v/v) for 1 day. After washing with tap water, the specimens were deparaffined in xylene and dehydrated in graded ethanol series (80–100%), and embedded in paraffin. The sample tissues were sectioned at 6–7 μm thickness and were stained with hematoxylin and eosin (H&E). The sections were examined under light microscopy (DMLA; Leica, Wetzlar, Germany).

### Statistical analysis

All data are presented as mean ± SE, and the statistical difference between each group was assessed by Kruskal–Wallis analysis with pairwise comparisons using a Tukey’s post-hoc test (GraphPad Prism, version 5.01; GraphPad). *P* < 0.05, *P* < 0.01, and *P* < 0.001 were considered statistically significant as specified for the measurement.

## Results

### Increased expression of type I collagen in hADSC conditioned media by F-CM in vitro

**Fig. 2:**
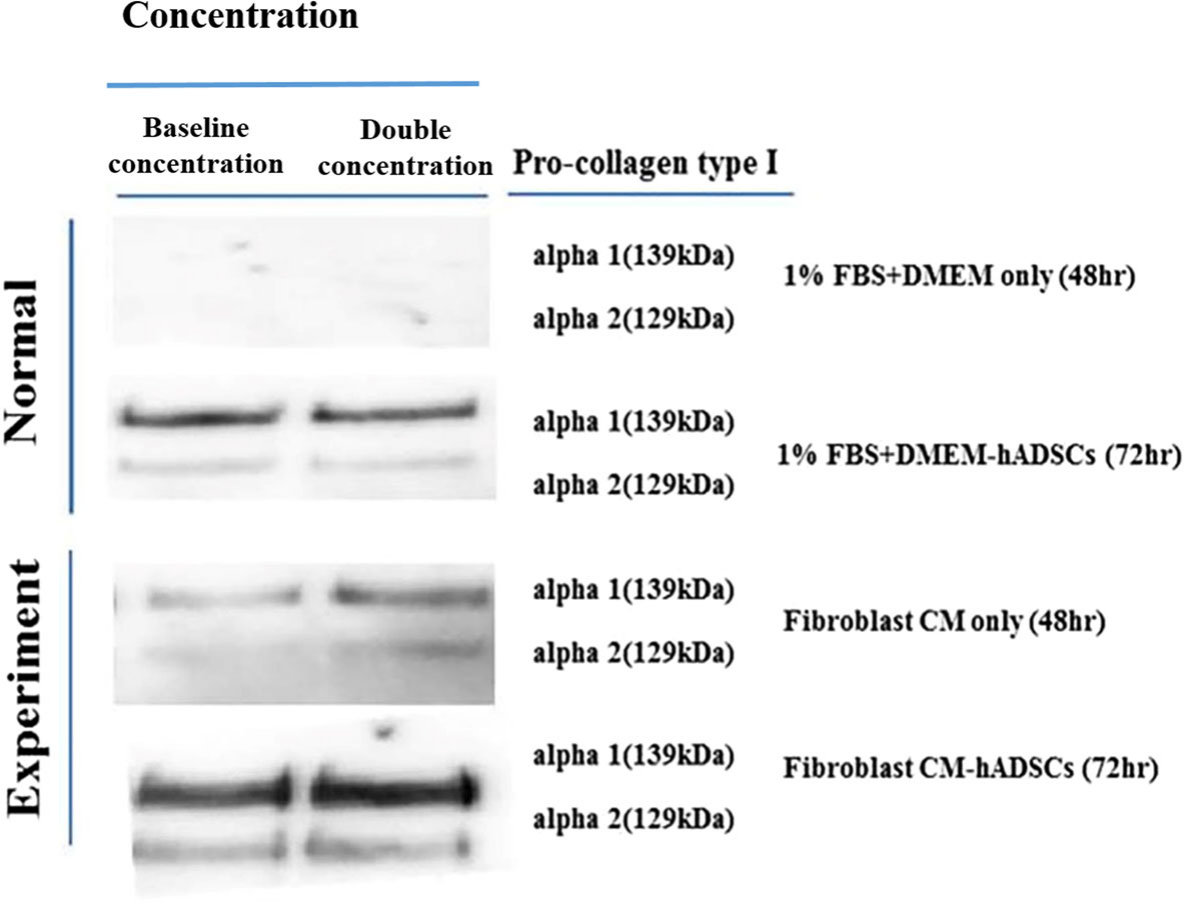
Comparison of pro-collagen type I protein levels for 1% FBS + DMEM only (24 h), 1% FBS + DMEM hADSCs (72 h), fibroblast CM only (24 h), and F-CM hADSCs (72 h): according to first or second density of DMEM and F-CM medium through western blot analysis; and in baseline concentration and double concentration DMEM and F-CM medium through western blot analysis. *CM* derived conditioned medium, *hADSC* human adipose-derived stem cell

**Fig. 3:**
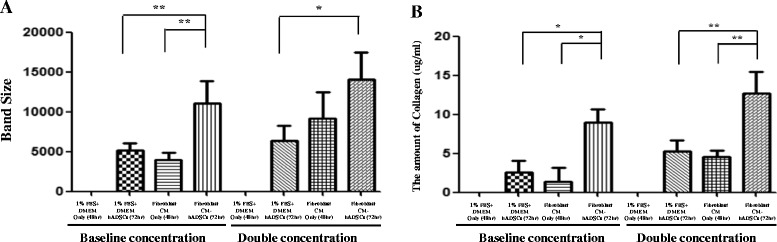
**a** Band intensities were significantly increased for type I pro-collagen protein expression shown at higher concentration of F-CM-treated hADSCs, when compared with 1% FBS + DMEM only, 1% FBS + DMEM ADSCs, or F-CM only after incubation for 72 h. **b** Amount of collagen (μg/ml) measured for each of the four groups in baseline concentration and double concentration normal and F-CM medium through Sircol assay. *P < 0.05, **P < 0.01. *CM* derived conditioned medium, *hADSC* human adipose-derived stem cell

To determine the expression of type I pro-collagen in F-CM for treated hADSC conditioned media, we treated the hADSC conditioned media with the following: 1% FBS + DMEM only media; 1% FBS + DMEM media conditioned with ADSC conditioned media; F-CM only media; and F-CM conditioned with ADSC conditioned media. Both with baseline concentration and double concentration concentrated media, expression of type I collagen by western blot analysis was significantly increased in F-CM-treated hADSC conditioned media compared with 1% FBS + DMEM-treated ADSC conditioned media after 72 h as well as F-CM-treated conditioned media for only 48 h (Fig. 2). Both in baseline and double concentration media, expression of type I collagen by western blot analysis was significantly increased in F-CM-treated hADSC conditioned media compared with 1% FBS + DMEM-treated ADSC conditioned media after 72 h as well as F-CM-treated conditioned media for only 48 h (Fig. 2). The band intensities were significantly increased for type I pro-collagen, and protein expression was higher for F-CM-treated hADSC conditioned media, when compared with 1% FBS + DMEM-only media, 1% FBS + DMEM-ADSC conditioned media, and F-CM-only media, after a 72-h incubation (Fig. 3a).

Moreover, the amount of total collagen measured by the Sircol assay indicated a similar pattern as western blot analysis occurring at about baseline concentration density (Fig. 3b).

### Characterization of hADSCs, HS27 cells, and F-CM hADSCs

hADSCs have been well characterized with cluster of differentiation (CD) markers and along with mesenchymal stem cells they express CD105, CD73, and CD90 and lack the expression of hematopoietic lineage markers c-kit, CD14, CD11b, CD34, CD45, CD79, CD19, and HLA-DR [24–27].

**Fig. 4:**
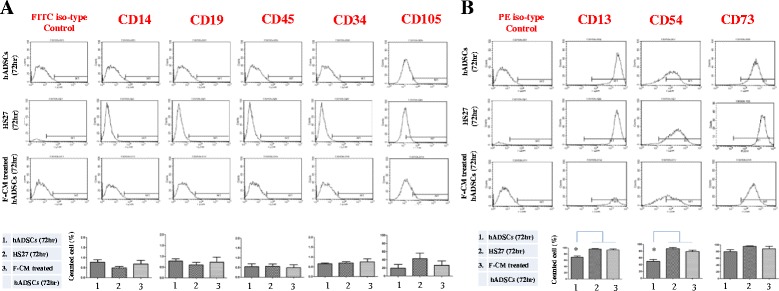
FACS analysis for characterization of differentiation of untreated hADSCs (72 h), HS27 cells (72 h), and F-CM-treated hADSCs (72 h) using cell surface makers: **a** CD14, CD19, CD45, CD34, and CD105; **b** CD13, CD54, and CD73. *F-CM* fibroblast-derived conditioned medium, *hADSC* human adipose-derived stem cell

As shown in Fig. 4a, b, hADSCs exhibited a fibroblast-like morphology. F-CM-treated hADSCs showed closer similarity to HS27 cells rather than hADSCs. In particular, significant similarity in expression of CD13 and CD54 was seen for F-CM-treated hADSCs and HS27 cells.

### Differentiation of hADSCs into fibroblast-like cells by F-CM in vitro

**Fig. 5:**
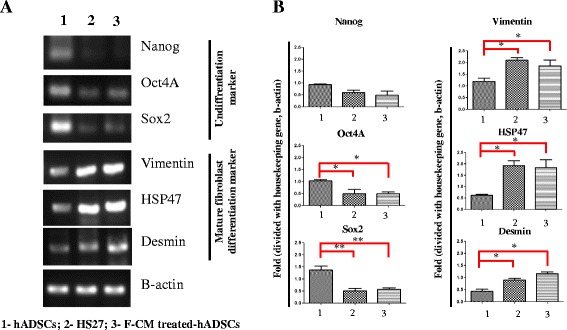
**a**, **b** mRNA levels of Nanog, Oct4a, and Sox2 as undifferentiated markers along with vimentin, HSP47, and desmin as mature fibroblast differentiation markers with RT-PCR in untreated hADSCs (72 h), HS27 cells (72 h), and F-CM-treated hADSCs (72 h). **a** semi-quantitative RT-PCR image for RNA levels of Nanog, Oct4a, and Sox2, vimentin, HSP47, and desmin. **b** the graph for relative comparision of each band thickness image in semi-quantitative RT-PCR image (**a**). *P < 0.05, **P < 0.01. *F-CM* fibroblast-derived conditioned medium, *hADSC* human adipose-derived stem cell

To confirm whether hADSCs induced by F-CM for 72 h were differentiated into fibroblast-like cells, FACS was performed for various surface markers. Both CD13 and CD54 levels were prominent on F-CM-treated hADSCs for 72 h and HS27 cells, but were significantly different from untreated hADSCs (Fig. 4a, b; *P* < 0.05). Furthermore, RT-PCR revealed the mRNA expression changes of various differentiation markers for F-CM-treated hADSCs compared with HS27 cells and untreated hADSCs. Nanog, Oct4A, and Sox2 are known to be high in undifferentiated cells, and vimentin, HSP47, and desmin were high in mature fibroblasts. Oct4A and Sox2 in untreated hADSCs showed significant increased expression rather than in the F-CM-treated hADSCs and HS27 cells. Conversely, vimentin, HSP47, and desmin message levels in untreated hADSCs were significantly lower than in both F-CM-treated hADSCs and HS27 cells (Fig. 5a, b; *P* < 0.05 and *P* < 0.01).

**Fig. 6:**
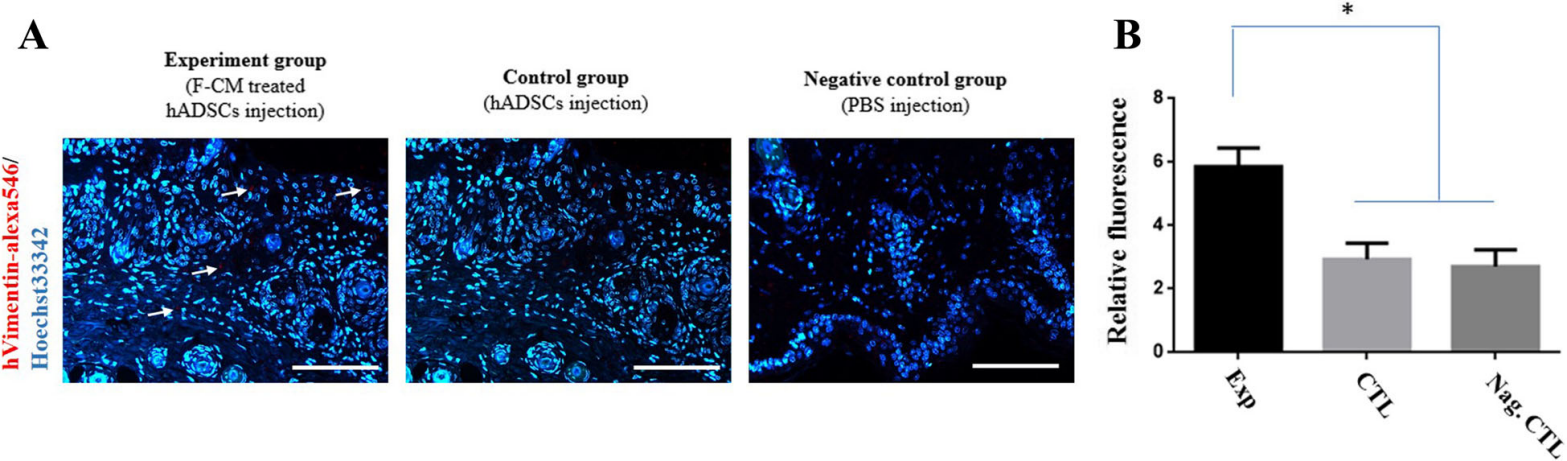
**a** Immunohistochemistry and **b** human vimentin localization in balb/c nude mice skin after injection of F-CM-treated hADSCs, untreated hADSCs, and PBS. Alexa-546 as secondary antibody and Hoechst 33342 as counterstain. *Arrows* indicate positive cells for immunofluorescence. *Scale bars* represent 100 μm. *P < 0.05. *CTL* control, *F-CM* fibroblast-derived conditioned medium, *hADSC* human adipose-derived stem cell, *PBS* phosphate-buffered saline

Immunohistochemistry was also performed in order to confirm whether F-CM-treated hADSCs transdifferentiated into fibroblasts. In brief, after F-CM-treated hADSCs and hADSCs were cultured according to protocol, each cell type was mixed with Cytodex microcarrier beads (Sigma-Aldrich) serving as a cell carrier. Next, the backs of balb/c nude mice were injected with the aforementioned mix of cells or PBS as control. At 2 weeks post implantation, the mice were sacrificed for biopsy and samples were embedded in paraffin. For immunohistochemistry analysis, we stained the samples with human induced vimentin as the first antibody, followed by Alexa 546 (red color) as the second antibody. Hoechst 33342 was used as the counterstain. The F-CM-treated hADSC-injected group (5.86 ± 0.49) presented a significantly increased obvious red spot compared with untreated injected hADSCs (2.93 ± 0.42) and the PBS group (2.70 ± 0.43) (Fig. 6a, b; *P* < 0.05).

### Mechanism of pro-collagen type I increase in F-CM-treated hADSCs in vitro

**Fig. 7:**
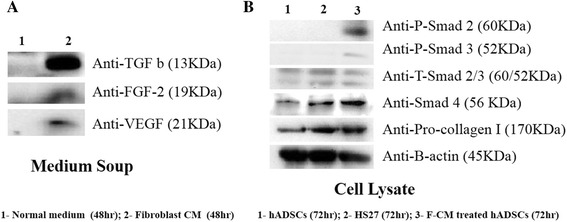
**a** Protein level comparison between normal medium (48 h) and F-CM (48 h) with anti-TGF-β, anti-FGF2, and anti-VEGF blotting. **b** For cell lysates, protein level comparison of untreated hADSCs (72 h), HS27 (72 h), and F-CM-treated hADSCs (72 h) through western blot analysis. *CM* conditioned medium, *hADSC* human adipose-derived stem cell

Figure 7a shows the molecules present in the F-CM medium soup. According to the figure, a mechanism emerges for production of pro-collagen type I in F-CM-treated hADSCs (Fig. 7a, b). Briefly, HS27 F-CM (DMEM containing 1% FBS and 1% P/S) was collected at 48 h of culture of HS27 cells. In comparison with unconditioned DMEM, the molecules in the two media types were subjected to anti-TGF-β, anti-FGF2, and anti-VEGF western blot analysis. F-CM had an intense expression of TGF-β and a low expression of FGF-2 and VEGF compared with the unconditioned medium. Figure 7b indicates a mechanism for production of pro-collagen type I by the smad signaling pathway. The hADSCs were then treated by F-CM for 72 h, as opposed to untreated hADSCs that were only cultured in regular DMEM for 72 h. For comparison, HS27 cells were also cultured for 72 h. From the western blot analysis, both phospho-smad2 and phospho-smad3 were detectable in the F-CM-treated hADSCs and not the ADSCs and HS27 cells. In addition, smad4 levels were higher in the three groups.

**Fig. 8:**
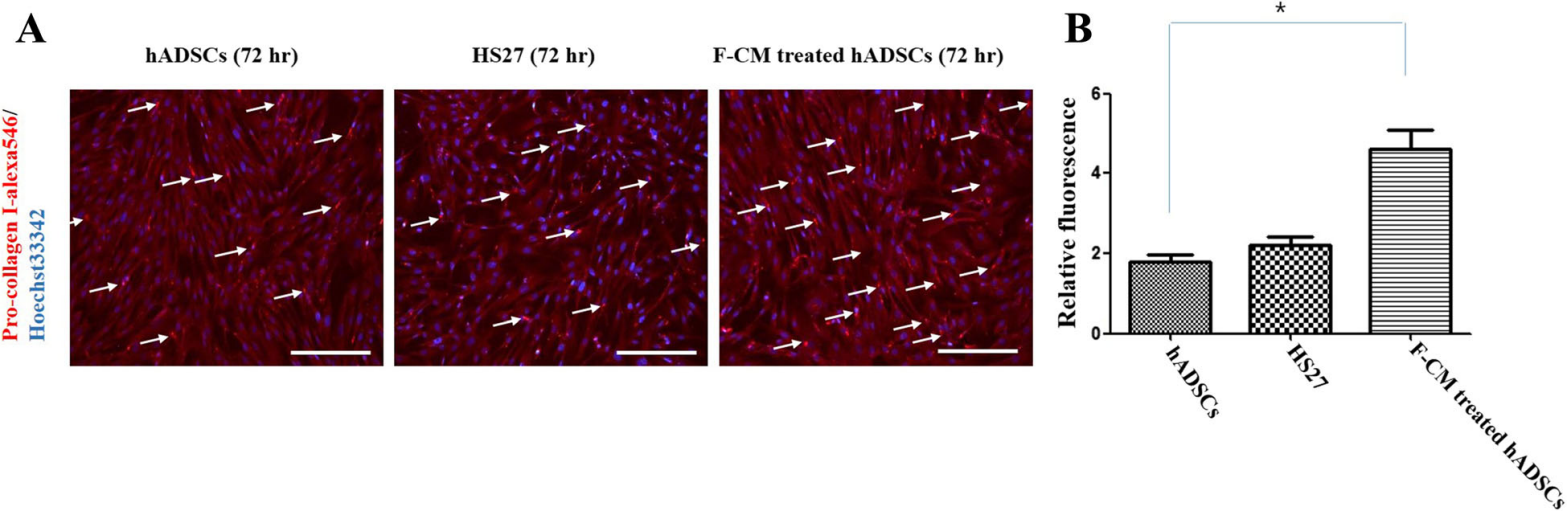
**a** Immunocytochemistry and **b** pro-collagen type I localization for untreated hADSCs (72 h), HS27 cells (72 h), and F-CM-treated hADSCs (72 h). *Arrows* indicate positive cells for immunofluorescence. *Scale bars* represent 100 μm. *P < 0.05. *F-CM* fibroblast-derived conditioned medium, *hADSC* human adipose-derived stem cell

Finally, F-CM-treated hADSCs exhibited a stronger expression for pro-collagen type I than the other groups. Moreover, F-CM-treated hADSCs (4.60 ± 0.70), untreated hADSCs (1.80 ± 0.25), and HS27 cells (2.20 ± 0.30) at 72 h were analyzed for levels of human pro-collagen type I with immunocytochemistry analysis from the in-vitro samples (Fig. 8a, b). The F-CM-treated hADSCs had a significantly strong expression of pro-collagen type as shown by the red staining compared with the untreated hADSCs, which showed little expression (*P* < 0.05).

### Wound healing on animals in vivo

**Fig. 9:**
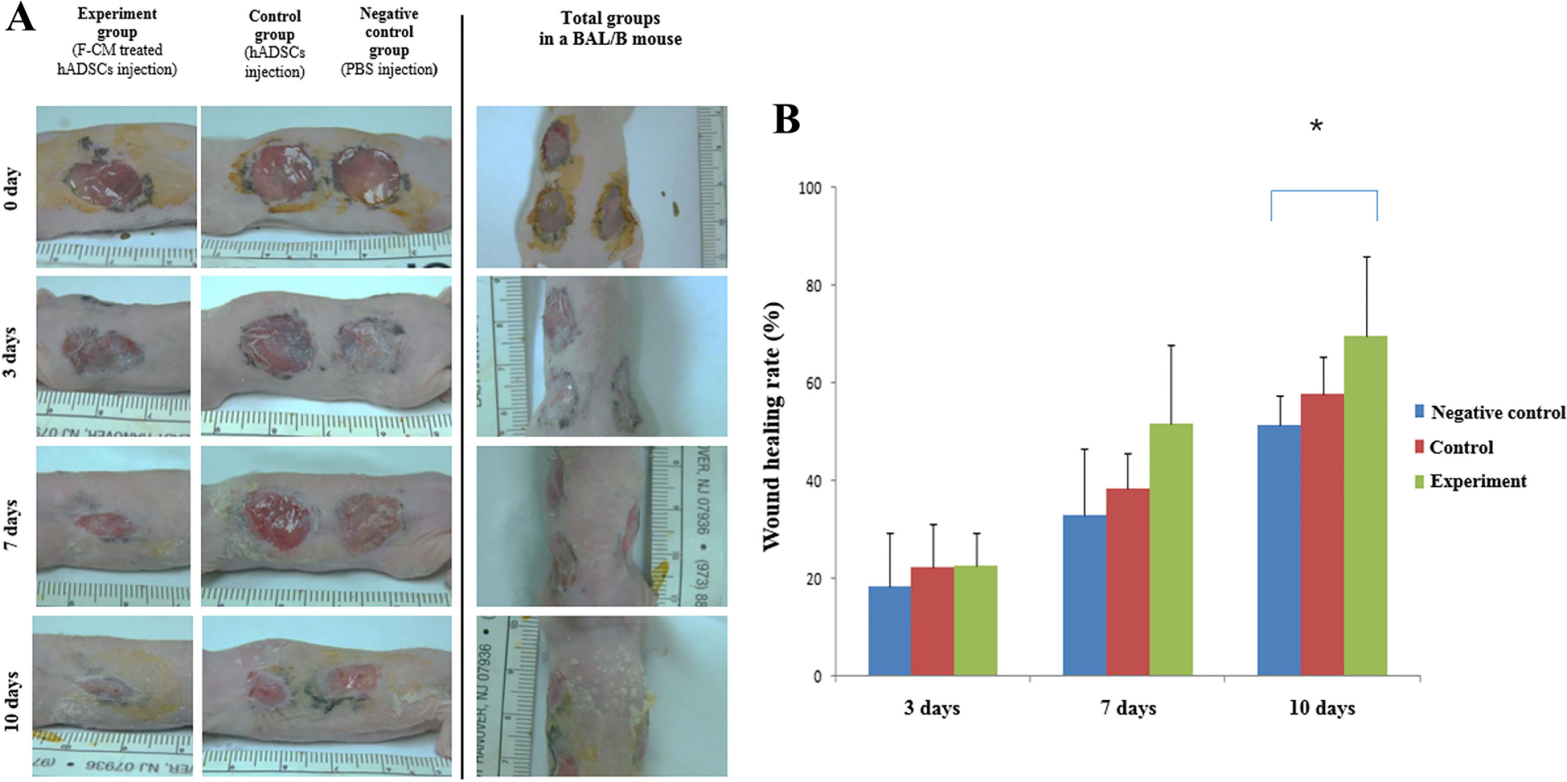
**a** Image for wound healing measurement and **b** wound healing rate percentages following injection of F-CM-treated hADSCs, untreated hADSCs, and PBS according to day post injection (0, 3, 7, and 10 days after cell implantation in balb/c nude mice skin). **P < 0.01. *F-CM* fibroblast-derived conditioned medium, *hADSC* human adipose-derived stem cell, *PBS* phosphate-buffered saline

To measure wound healing in vivo, a full-thickness excision wound model was created by inflicting a 10-mm circular wound on the back of balb/c nude mice (Fig. 9). The F-CM-treated hADSCs were injected into the wound sites and separately untreated hADSCs were injected as control cells; PBS injection without cells served as a negative control. At 3 days post cell implantation, all three groups showed a similar pattern with minimal wound healing evident. At 7 days, however, grafts of the F-CM-treated hADSC injection group had integrated into the surrounding skin, and their color was similar to normal skin. In the untreated hADSC and PBS groups, only small parts of grafts had integrated into the wound with little scarring and the color of the graft was darker than that of the normal skin. At 10 days, the F-CM-treated hADSC injection group exhibited accelerated wound contraction and reepithelialization, showing significant reduction in wound size compared with the PBS injection group (*P* < 0.01).

### Histological evaluation

**Fig. 10:**
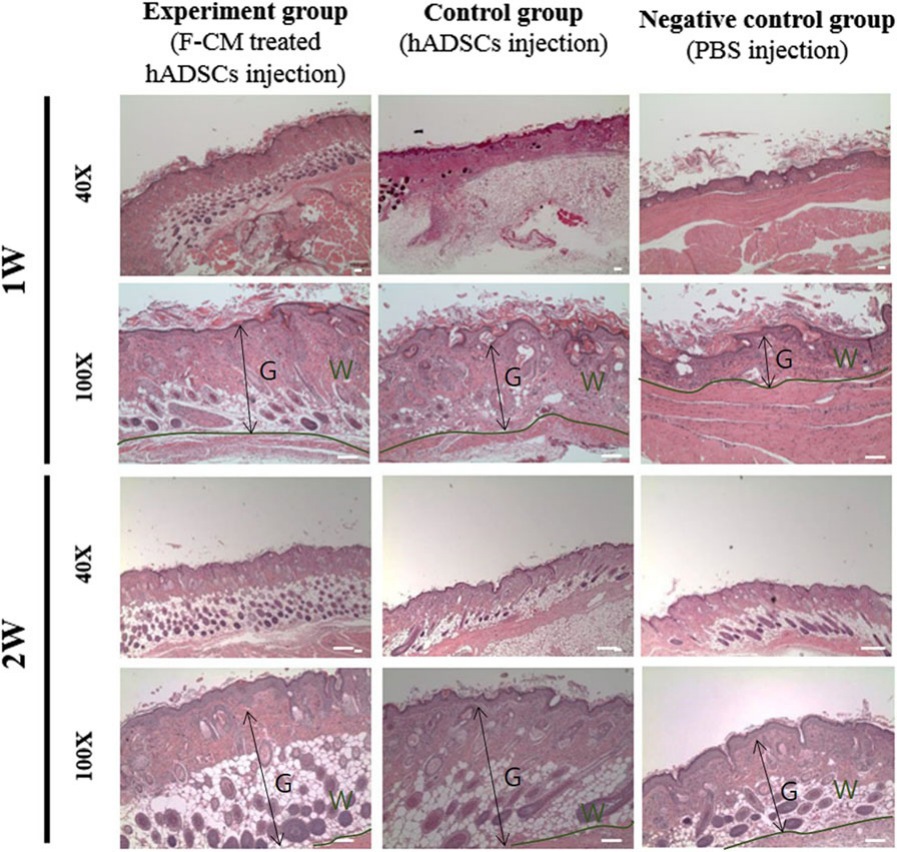
Histological evaluation of wound samples from F-CM-treated hADSC, untreated hADSC, and PBS injected animals at 1 week (*1 W*) and 2 weeks (*2 W*) after cell implantation in balb/c nude mice skin using H&E staining. Magnification: 40×, 100×. *F-CM* fibroblast-derived conditioned medium, *hADSC* human adipose-derived stem cell, *PBS* phosphate-buffered saline, *G* Granulation tissue, *W* Wounded area

**Fig. 11:**
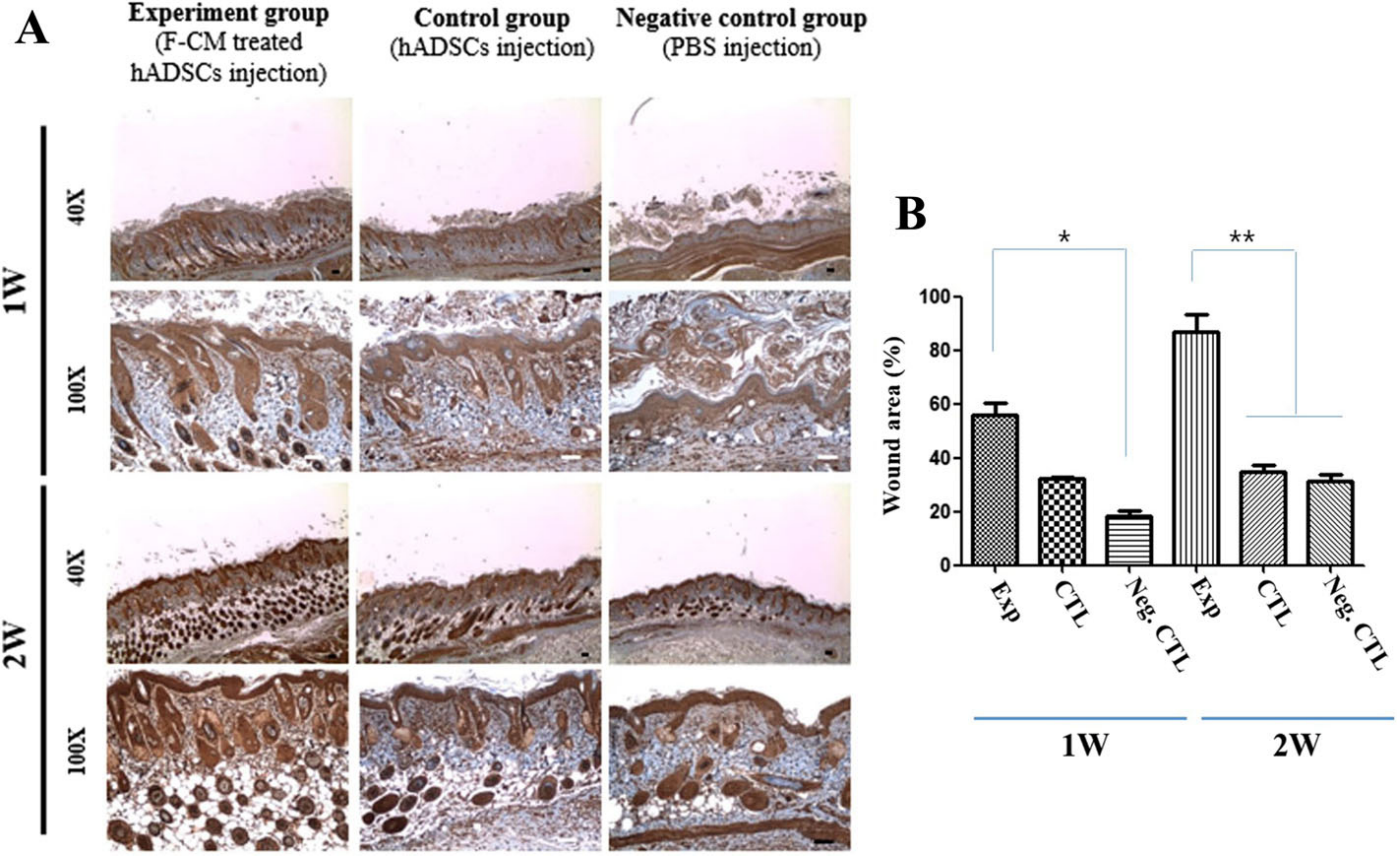
**a** Histological evaluation and **b** F-CM-treated hADSC, untreated hADSC, and PBS injected animals at 1 week (*1 W*) and 2 weeks (*2 W*) after cell implantation in balb/c nude mice skin with anti-collagen type I staining. Magnification: 40×, 100×. *P < 0.05, **P < 0.01. *CTL* control, *F-CM* fibroblast-derived conditioned medium, *hADSC* human adipose-derived stem cell, *PBS* phosphate-buffered saline

To evaluate the histology of wound healing in vivo, we obtained samples at 1 and 2 weeks after cell implantation from the full-thickness excision wounded back. The samples were paraffin embedded, sectioned, and H&E stained along with undergoing immunohistochemical analysis. As shown in Fig. 10, samples from the F-CM-treated hADSC injection group indicated merging of the grafts with the border of dermis, leading to an unclear boundary compared with the other two groups at week 1 of grafting (Fig. 10). Also, both the untreated hADSC and PBS injection groups showed rich fibrosis and follicle disappearance caused by long-term wound healing. For the collagen type I patterns, for all three groups, a similar pattern was seen in which collagen type I was stained a dark brown color, but there was no staining detected in the dermis areas (Fig. 11a).

At 2 weeks post implantation of cells, the F-CM-treated hADSC injection group wound samples showed an increase in dermal thickness with little fibrosis. In addition, the follicle densities were no different between the wounded skin and the normal skin (Fig. 10). However, for the wound samples in the untreated hADSC and PBS injection groups, there was a slight reduction in the follicle density and a slight shrinkage in the structure of dermis. In general, when inflammation is very severe and wound healing is lengthy, there is abundant fibrosis. A paucity of fibrosis means rapid wound healing, leading to a normal skin structure. Abundant fibrosis causes dense scarring, and the dermis structure will disappear following a reduction in follicle densities. An increase in levels of collagen type I in the F-CM-treated hADSC injection group (82.12 ± 2.31) was significantly evident with intense staining in brown rather than the untreated hADSC (38.29 ± 1.26) and PBS (36.82 ± 1.39) injection groups (Fig. 11a, b; *P* < 0.05). The other two groups only showed blue staining, indicating minimal or no increase in collagen type I levels in the dermis.

## Discussion

Stem cells are a subject of intense research in the cell replacement and tissue engineering fields. There have been many reports of stem cells for regeneration of wounded tissue using specific differentiated cells. However, we did not come across a study where stem cells were transdifferentiated into fibroblasts in order to accelerate healing. Fibroblasts are very important in the promotion of wound healing, and we recognized that fibroblasts are ubiquitous cells in wound areas undergoing healing, but their numbers may not be adequate to promote optimal healing in normal circumstances. Therefore, we tried to differentiate hADSCs into fibroblast-like cells and tested their potency in mediating wound healing.

In the present study, we demonstrated that hADSCs could be differentiated into fibroblast-like cells by culturing them in F-CM. By FACS analysis, we demonstrated that the stem cell properties of hADSCs allowed them to differentiate into fibroblast-like cells. Compared with other MSCs, hADSCs can be harvested easily from patients and can be cultured and expanded rapidly. In addition, long-term cultured ADSCs retain their mesenchymal pluripotency [16]. In this study, we confirmed that untreated hADSCs not only expressed characteristic surface markers (positive for CD105, CD13, CD54, and CD73; negative for CD149, CD19, CD45, and CD34), but were also significantly different from HS27 cells and F-CM-treated hADSCs. We aim to validate that hADSCs would differentiate into fibroblast-like cells with F-CM in order to stimulate wound healing in a skin defect model. With hADSCs harvested from human adipose tissue, we induced differentiation of hADSCs into fibroblast-like cells by F-CM treatment to meet the requirement of massive fibroblast numbers for thick skin wound healing. To confirm that hADSCs induced by F-CM for 72 h had differentiated into fibroblast-like cells, FACS analysis was performed; this showed that F-CM-treated hADSCs and HS27 cells had similar surface markers, and those were significantly different from untreated hADSCs (Fig. 4a, b). In addition, RT-PCR and immunohistochemistry also indicated increased expression of matured fibroblast-like markers in F-CM-treated hADSCs, an obvious result of differentiation (Fig. 5a, b).

Human dermal skin fibroblasts play key roles in wound healing by secretion of type I collagen and cytokines [28, 29]. We confirmed the expression of type I pro-collagen protein in F-CM-treated hADSC conditioned media with western blot analysis as well as the expression of total collagen protein level with the Sircol assay. Figures 2 and 3a indicated that the band size was significantly increased for type I pro-collagen, indicating a sizable increased expression in F-CM-treated hADSC conditioned media (baseline concentration, double concentration) at 72 h after treatment. In addition, the amount of total collagen also showed a similar result, with a significantly increased amount in F-CM-treated hADSC conditioned media (baseline concentration, double concentration) at 72 h post treatment (Fig. 3b).

In recent studies, transplantation of BMSCs has been reported to promote the healing process due to the cells’ capacity to differentiate into the skin epidermis and appendages, thus mediating dermal regeneration [30]. Also, several studies have recently demonstrated accelerated rates of wound closure after transplantation of BMSCs, mesenchymal stem cells, and ADSCs [31, 32]. Moreover, in cutaneous wounds, partial or whole epidermis is destroyed, but the vascular structure is not damaged. Unaltered ADSCs exert only a moderate therapeutic potential compared with fibroblast-like differentiated ADSCs. These unaltered ADSCs can also differentiate into endothelial cells and smooth muscle cells, which contribute to about 9% of the ADSC-mediated angiogenesis, and as such unaltered ADSCs induce a beneficial effect that is predominantly mediated by a paracrine mechanism, with direct in-vivo differentiation playing a minor role [33].

To study the effect of F-CM-treated hADSCs in wounded skin in vivo, we intradermally injected F-CM-treated hADSCs and untreated hADSCs into the backs of balb/c nude mice in a full-thickness excision wound model (Fig. 9). The F-CM-treated hADSC injection group showed accelerated wound contraction and reepithelialization reduced the wound size compared with the unaltered hADSC and PBS injection groups at 10 days. To evaluate the histology of wound healing in vivo*,* we sacrificed mice sequentially at 1 and 2 weeks post implantation. With H&E staining, samples from the F-CM-treated hADSC injection group indicated an increased wound healing pattern at 2 weeks (Fig. 10). Immunohistochemical analysis indicated a sizable increase in staining of collagen type I with intense brown staining clearly observed in the F-CM-treated hADSC injection group compared with the other two groups (Fig. 11).

Secreted factors FGF, TGF-β1, and PDGF promote synthesis, deposition, and organization of new ECM collagen, fibronectin, and additional FGF, and TGF-β, which all contribute to wound healing, cell signaling, and tissue remodeling [34, 35]. The TGF-β signaling pathway plays an important role in each of these processes. The TGF-β1 signaling mechanism functions through the TGF-β type I (TbRI) and TGF-β type II (TbRII) transmembrane serine/threonine protein kinase receptors. Upon TGF-β1 binding to its type II receptor, TbRI is recruited to TbRII where it forms a ligand–receptor heterotetrameric complex [36, 37]. Under physiological conditions, TLP binds the type II receptor even when the pathway has been previously activated by TGF-β1, and the type II receptor is constitutively active. It transphosphorylates and activates the type I receptor, whose direct substrates are Smad2 and Smad3. Phosphorylation of receptor-activated Smads (R-Smads) leads to formation of complexes with the common mediator Smad (Co-Smad), which are then imported to the nucleus. Nuclear Smad oligomers bind to DNA and associate with transcription factors to regulate expression of target genes [38, 39]. In the process of tissue fibrosis, TGF-β1 is likely to facilitate the expression of the ECM genes for an increase in the synthesis and deposition of collagen, fibronectin, and proteoglycan [40].

We demonstrated a mechanism for upregulation of pro-collagen type I in F-CM-treated hADSCs with F-CM containing TGF-β, FGF2, and VEGF cytokines (Fig. 7). F-CM-treated hADSCs exhibited increased pro-collagen type I expression via activated phospho-smad2, phospho-smad3, and smad4, not seen with the other groups of cells. hADSCs treated by F-CM, untreated hADSCs, and HS27 cells for 72 h were also analyzed for human collagen type I levels with immunocytochemistry in vitro (Fig. 8). In future studies, we plan to compare the levels of F-CM-treated hADSC fibroblasts and endogenous fibroblasts without ADSC stimulation in the different wound healing scenarios.

In conclusion, our group aimed to differentiate F-CM-treated hADSCs into fibroblast-like cells and confirmed the efficiency of differentiated cells in promoting collagen type I synthesis in a wound healing model. With potential applications in the clinic, our study is the first research of its kind with differentiated hADSCs being applied for treating full-thickness skin wounds.

## Conclusions

F-CM contains a variety of factors known to be important in healing skin wounds. Our study revealed that F-CM had factors promoting collagen synthesis in hADSCs as well as transdifferentiation of hADSCs into fibroblast-like cells. These cells could stimulate wound healing in a skin defect model. After hADSC transdifferentiation by F-CM, the changes in expression of target molecules involved the use of western blot analysis, Sircol collagen assay for collagen increase, as well as FACS and RT-PCR. We confirmed an increased expression of type I pro-collagen through smad 2/3 protein upregulation in F-CM-treated hADSCs. In vivo*,* we verified a significant increase in the level of wound healing in balb/c nude mice skin, implanted with F-CM-treated hADSCs. Our findings may contribute to applications in stem cell therapy and regenerative medicine. Furthermore, these findings may argue for using cell therapy without gene or protein modification.

## Abbreviations

ECM: Extracellular matrix
F-CM: Fibroblast-derived conditioned medium
hADSC: Human adipose-derived stem cell
hADSC-CM: Human adipose-derived stem cell-derived conditioned medium
